# Activin B can signal through both ALK4 and ALK7 in gonadotrope cells

**DOI:** 10.1186/1477-7827-4-52

**Published:** 2006-10-13

**Authors:** Daniel J Bernard, Katharine B Lee, Michelle M Santos

**Affiliations:** 1 Center for Biomedical Research, Population Council and The Rockefeller University, 1230 York Ave., New York, NY 10021, USA; 2 Department of Pharmacology and Therapeutics, McGill University, 3655 Promenade Sir William Osler, Montréal, Québec H3G 1Y6, Canada

## Abstract

**Background:**

Activins stimulate pituitary FSH synthesis via transcriptional regulation of the FSHbeta subunit gene (Fshb). Like other members of the TGFbeta superfamily, these ligands signal through complexes of type I and type II receptor serine/threonine kinases. The type I receptors, or activin receptor-like kinases (ALKs), propagate intracellular signals upon ligand binding and phosphorylation by associated type II receptors. ALK4 is generally regarded as the type I receptor for activins; however, recent data suggested that activin B and AB might also signal through ALK7. Here, we examined a role for ALK7 in activin B-regulated Fshb transcription.

**Methods:**

We analyzed ALK7 mRNA expression in immortalized gonadotrope cells, LbetaT2, and adult murine pituitary by RT-PCR. We next transfected LbetaT2 cells with wild-type and kinase-deficient (Lys to Arg, KR) forms of ALK4 and ALK7 and examined the effects of these receptors on activin A and B stimulated Fshb promoter-reporter activity. Cells were also transfected with constitutively active (Thr to Asp, TD) forms of the receptors and their effects on endogenous Fshb mRNA levels and phosphorylation of transfected Smad2/3 were measured by RT-PCR and Western blot, respectively. Finally, we measured ALK4(TD) and ALK7(TD) stimulation of Fshb transcription when endogenous Smad3 levels were depleted using short hairpin RNAs.

**Results:**

ALK7 mRNA was expressed in LbetaT2 cells and pituitary gland. Transfection of ALK4 cDNA potentiated the effects of both activin A and activin B on Fshb promoter-reporter activity in LbetaT2 cells. In contrast, ALK7 transfection selectively potentiated activin B's effects. Transfection of ALK4(KR) and ALK7(KR) partly inhibited basal and activin B-stimulated reporter activity, whereas ALK4(TD) and ALK7(TD) potently stimulated the Fshb promoter and endogenous mRNA levels. Transfection of both ALK4(TD) and ALK7(TD) stimulated Smad2/3 phosphorylation, and the effects of both receptors on Fshb promoter activity were inhibited by depletion of endogenous Smad3 protein levels.

**Conclusion:**

These data suggest that immortalized gonadotropes express ALK7 and that activin B can signal through this receptor to stimulate Fshb transcription. The relative roles of endogenous ALK4 and ALK7 receptors in mediating activin B's effects in these cells have yet to be determined.

## Background

Activins were first isolated from porcine follicular fluid during the purification of the inhibins and were shown to have potent and selective stimulatory effects on pituitary FSH secretion [1,2]. Their effects are largely mediated via up-regulation of FSHβ (*Fshb*) subunit gene transcription [3-5]. Unlike the inhibins which function as gonadally-derived endocrine hormones, activins in the circulation are bound to follistatins and are therefore biologically inactive [6,7]. However, the activin subunits are expressed in adult pituitary gland, suggesting that activins can be produced locally and may function as paracrine or autocrine regulators of FSH production by gonadotropes cells. Indeed, the activin βB subunit (INHBB) is produced by rat gonadotropes *in vivo *and immortalized murine gonadotrope cells (LβT2) [5,8,9], and its immunoneutralization inhibits FSH release from rat pituitary cell cultures [10]. In contrast, the activin βA subunit (INHBA) is expressed throughout the pituitary, though not in LβT2 cells, and INHBA subunit bio-neutralizing antibodies have no effect on FSH secretion [8-10]. These data suggest that activin B (a homodimer of INHBB subunits) is the physiologically relevant activin family member in the pituitary.

TGFβ superfamily proteins produce their effects in target cells by binding complexes of type I and type II receptor serine/threonine kinases [11,12]. For activins, the ligand binding type II receptors are ACVR2A and ACVR2B [13,14]. Once bound, these receptors phosphorylate the type I receptor, activin receptor-like kinase 4 (ALK4; ACVR1B), in a juxtamembrane domain called the GS box [15,16]. This activates the type I receptor, allowing it to phosphorylate intracellular effectors such as the Smads. A second type I receptor, ALK2 (also known as ACVR1), was shown to bind activin A, but does not appear to transduce the ligand's intracellular signals [15,17,18]. Thus, ALK4 has conventionally been considered the only type I receptor for the activins.

This notion was recently challenged by the observation that another type I receptor, ALK7 (ACVR1C), could propagate activin B and activin AB signals in a murine pancreatic β cell line [19]. Interestingly, the receptor appeared insensitive to activin A, suggesting that unique features of the INHBB subunit [20] permit its association with this receptor. Given activin B's purported role in FSH regulation, we examined whether the ligand could signal through ALK7 to stimulate the *Fshb *subunit gene in gonadotropes.

## Methods

### Ligands and constructs

Human recombinant (rh-) activin A and activin B were purchased from R&D systems (Minneapolis, MN). Rat ACVR1 (ALK2)-HA and rat ACVR1B (ALK4)-HA expression vectors were generously provided by Dr. T. Woodruff (Northwestern University, Evanston, IL). Wild-type and constitutively active (T194D) human ACVR1C (ALK7)-myc/His constructs were gifts from Dr. C. Peng (York University, Toronto, Canada). Constitutively active ALK4(T206D)-HA, kinase-deficient ALK4(K234R)-HA, and kinase-deficient ALK7(K222R)-myc/His were generated by site-directed mutagenesis. Human Flag-Smad2 and human Flag-Smad3 were provided by Dr. E. Roberston (Oxford University, UK) and Dr. Y. Chen (Indiana University), respectively. All expression constructs contained the CMV promoter, except for Flag-Smad2, which was in a modified pCAGGS vector [21,22]. The -1990/+1 m*Fshb*-luc reporter construct and *Smad3 *shRNA vector were described previously [3].

### Cell culture, transfections, and luciferase assays

Immortalized mouse gonadotrope LβT2 cells were provided by Dr. Pamela Mellon (University of California, San Diego). Cells used in luciferase assays were seeded in 24-well plates and transfected with Lipofectamine 2000 (Invitrogen, Carlsbad, CA) as previously described [3,22]. Luciferase assays were performed on a Luminoskan Ascent luminometer (Thermo Labsystems, Franklin, MA) using standard reagents. All treatments were performed in duplicate or triplicate, and each experiment repeated two or three times as indicated. For RNA analysis of cells transfected with constitutively active receptors, cells were seeded in 6-well plates and transfected for 6 h with 1 μg of expression plasmid using Lipofectamine/Plus (Invitrogen). After 24 h incubation in growth medium, total RNA was isolated using Trizol (Invitrogen).

### Reverse transcriptase PCR

*ALK7 *and *Fshb *mRNA expression in adult male C57BL6/J mouse pituitary, adult male CD-1 mouse brain and LβT2 cell DNased total RNA were examined by RT-PCR using the following primer sets and previously described methods [3]: *ALK7*. for, ATGACCCCAGCGCGCGGCTCCGCACT; *ALK7*. rev, CTTCCTGTATGTGCACTGGCGGTCCT; *Fshb*. for, ATGAAGTTGATCCAGCTTTG; *Fshb*. rev, CATTTCACTGAAGGAGCAGT. RNA was extracted using Trizol.

### Immunoblot

LβT2 cells in 6-well plates were transfected for 6.5 hr with 0.9 μg/well ALK4(TD) or ALK7(TD) along with 2 μg/well pcDNA3, Flag-Smad2, or Flag-Smad3 using Lipofectamine/Plus (Invitrogen). After approximately 18 h, whole cell extracts were prepared in RIPA buffer. Equivalent amounts of protein (20 μg/lane) were separated by SDS-PAGE on a 7% Tris acetate gel (NuPage, Invitrogen) and transferred to a Protran nitrocellulose filter (Schleicher & Schuell, Keene, NH). The blot was probed with the following antibodies using standard techniques: phospho-Smad2 (Cell Signaling Technology, Danvers, MA), phospho-Smad3 (gift of Dr. M. Reiss, UMDNJ-RWJMS), Flag M2 and β-actin (Sigma, St. Louis, MO) HRP conjugated secondary antibodies were from Biorad (Hercules, CA) and ECL Plus reagents from GE Healthcare.

### Statistics

Treatments were performed in duplicate or triplicate, and each experiment repeated two or three times as indicated in the figure legends. Replicates within individual experiments were averaged and these values used in statistical analyses. Effects of the different manipulations were assessed with two-way analyses of variance (ANOVA) as described in the Results, and post-hoc comparisons of significant main effects or interactions were made using Fisher's LSD procedure. In all cases, significance was assessed relative to *p *< 0.05.

## Results

### ALK7 is expressed in pituitary gland and LβT2 cells

**Figure 1:**
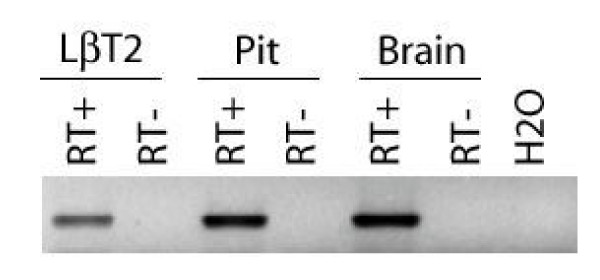
**ALK7 is expressed in LβT2 cells**. *ALK7 *mRNA expression was analyzed by RT-PCR in adult mouse pituitary and brain, and in LβT2 cell RNA. RT+ and RT- indicate that reverse transcriptase enzyme was included or omitted, respectively, during the cDNA synthesis step. The H_2_O sample confirmed that none of the reagents were contaminated.

We used RT-PCR to examine *ALK7 *mRNA expression in adult murine pituitary gland and immortalized LβT2 gonadotrope cells. ALK7 was first identified in rat brain [23,24]. Therefore, adult murine brain was used as a positive control. *ALK7 *mRNA was detected in all three samples (Fig. 1).

### ALK7 potentiates activin B signaling in LβT2 cells

**Figure 2:**
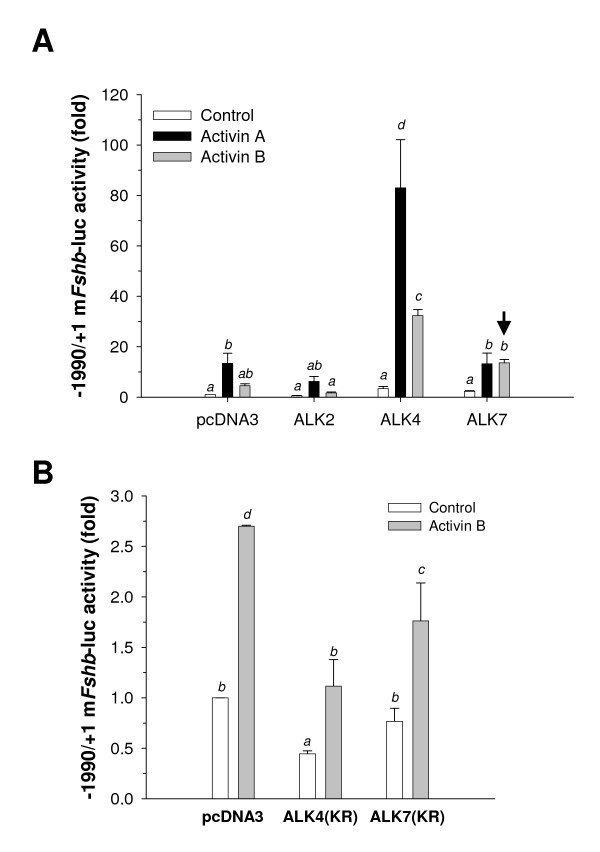
**ALK7 can potentiate activin B signaling**. A, LβT2 cells seeded in 24-well plates were transfected with 450 ng/well promoter-reporter and 200 ng/well of the indicated receptor expression vector. Cells were then treated with 25 ng/ml activin A or B in serum-free medium for 24 h. Cell lysates were collected and assayed for luciferase activity. Data are presented as fold difference from the control condition (pcDNA3, Control) set to 1 and represent the mean (± s.d.) of three independent experiments performed in duplicate. The arrowhead shows the potentiation of the activin B response in the presence of ALK7. B, Cells were transfected with kinase-deficient forms (KR) of ALK4 and ALK7 alone and treated with activin B as described in A. Data are from two independent experiments with treatments performed in triplicate. In both panels, bars with different letters differed significantly from one another.

Next, we transfected LβT2 cells with a murine *Fshb *promoter-reporter construct [3] along with expression vectors encoding wild-type ALK2, ALK4 or ALK7, followed by treatment with 25 ng/ml activin A or activin B for 24 h (Fig. 2A). Two-way ANOVA showed significant effects of ligand and receptor, and a significant interaction between the two variables. Post-hoc analysis revealed that activin A was more potent than activin B, as we have seen previously [9], and that ALK4 transfection had the most significant stimulatory effect of the three receptors examined. ALK7 differed significantly from ALK2-, but not pcDNA3-transfected cells. Pair-wise comparisons of the significant interaction showed that in the presence of transfected ALK4, the activity of both ligands was increased, though activin A was still more potent than activin B. ALK7 transfection had no effect on the activin A response, but enhanced activin B activity (Fig. 2A, *arrowhead*); however, the potentiation with exogenous ALK7 was less than that observed with ALK4. In the context of the entire analysis, ALK2 did not have a statistically significant effect. However, when the analysis was restricted to ALK2- versus pcDNA3-transfected cells (i.e., with data for ALK4 and ALK7 omitted), ALK2 significantly suppressed reporter activity (main effect of receptor) and inhibited the activin A response relative to control.

### Kinase-deficient forms of ALK4 and ALK7 inhibit basal and activin B regulated transcription

Previous data suggest that endogenous activin B signaling may regulate "basal" *Fshb *expression in LβT2 cells [5,9]. As an additional means to assess type I receptor function, we transfected expression constructs encoding kinase-deficient forms of ALK4 (K234R) and ALK7 (K222R) and then treated cells with activin B. These receptors can bind ligand, but do not propagate intracellular (Smad-dependent) signals and therefore act as dominant-negative regulators [25,26]. Two-way ANOVA revealed significant effects of ligand, receptor, and the interaction. Post-hoc comparisons showed that overall both ALK4(KR) and ALK7(KR) significantly diminished *Fshb *reporter activity (Fig. 2B). Pair-wise comparisons of the interaction showed that ALK4(KR) diminished basal reporter activity, and both receptors significantly inhibited the effects of activin B.

### ALK7 stimulates *Fshb *expression through a Smad-dependent pathway

**Figure 3:**
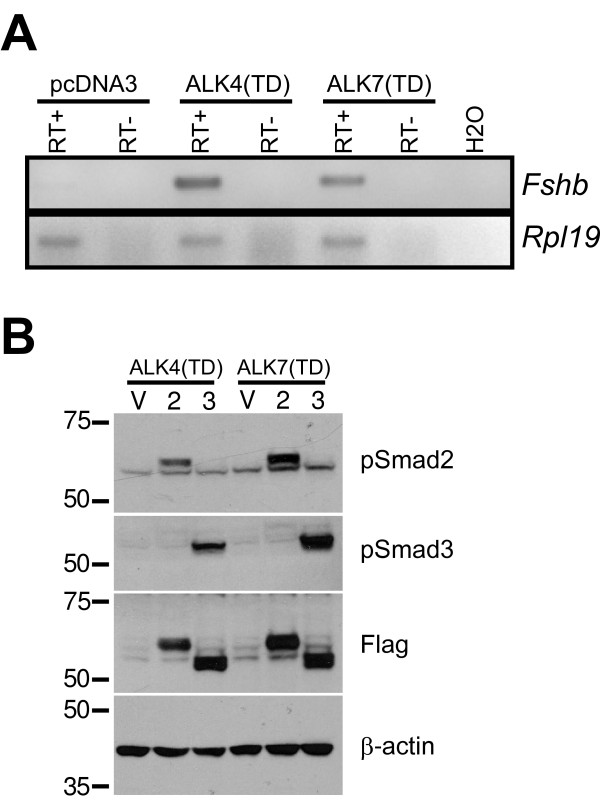
**Constitutively active forms of ALK4 and ALK7 stimulate *Fshb *mRNA expression and Smad2/3 phosphorylation**. A, LβT2 cells were transfected as described with constitutively active (TD) forms of ALK4 and ALK7. RNA was isolated and *Fshb *mRNA levels measured by RT-PCR. *Rpl19 *was used as a loading control. B, LβT2 cells in 6-well plates were transfected with ALK4(TD) or ALK7(TD) along with pcDNA3 (V), Flag-Smad2 (2), or Flag-Smad3 (3). Whole cell protein lysates were subjected to immunoblotting with the indicated antibodies. Molecular weight markers (in kDa) are indicated at the left.

**Figure 4:**
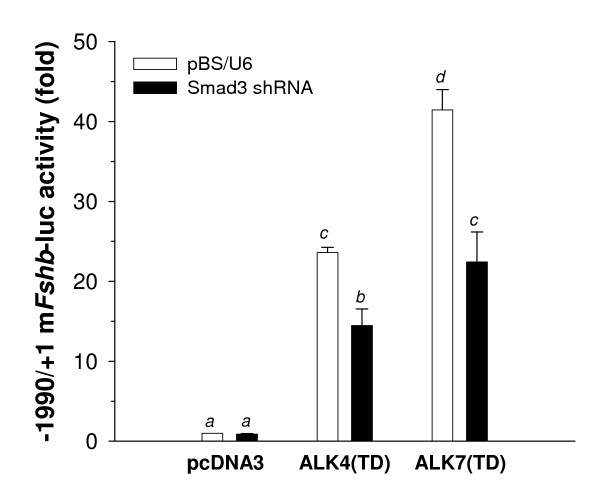
**Knockdown of Smad3 inhibits ALK4(TD) and ALK7(TD) stimulated transcription**. LβT2 cells were transfected as described with ALK4(TD) or ALK7(TD) in the presence of a *Smad3 *shRNA expression vector or empty vector (pBS/U6). Luciferase activity from the *Fshb *reporter was measured 24 hr post transfection. Data are from three independent experiments with treatments performed in triplicate. Bars with different letters differed significantly from one another.

To confirm that signaling events downstream of ALK7 regulate endogenous *Fshb *expression, we transfected LβT2 cells with constitutively active forms of ALK4 (T206D) [26] and ALK7 (T194D) [25]. We previously showed that ALK4(TD) stimulated *Fshb *expression in these cells in the absence of exogenous ligand or co-transfected type II receptors [3]. Here, both transfected ALK4(TD) and ALK7(TD) stimulated endogenous *Fshb *mRNA levels (Fig. 3A) and murine *Fshb *promoter-reporter activity (Fig. 4, see white bars).

The L45 loops of ALKs 4, 5, and 7 are identical and it is through this sub-domain that these three receptors interface with the intracellular signaling proteins Smads 2 and 3 [27]. We first confirmed that the two receptors could stimulate Smad2 and Smad3 phosphorylation in LβT2 cells. Cells were transfected with ALK4(TD) or ALK7(TD) and Flag-Smad2 or Flag-Smad3. Immunoblots showed that both Smads were phosphorylated by both receptors (Fig. 3B). Smads transfected in this manner in the absence of constitutively active receptors were not phosphorylated (data not shown).

We previously showed that ALK4(TD) signals through both of these Smads to regulate murine *Fshb *[3]. To determine if ALK7(TD) similarly uses a Smad-dependent pathway in these cells, we depleted intracellular Smad3 protein levels with a validated *Smad3 *shRNA [3] and measured -1990/+1 m*Fshb*-luc activity in response to transfected ALK4(TD) or ALK7(TD) (Fig. 4). Two-way ANOVA showed a significant effect of receptor, with ALK7(TD) stimulating reporter activity more than ALK4(TD), as well as a significant inhibitory effect of the Smad3 knock-down. Pair-wise comparisons of the significant interaction showed that transfection of the *Smad3 *shRNA inhibited the effects of both receptors to comparable extents [39 and 46% for ALK4(TD) and ALK7(TD), respectively], without affecting basal activity of the promoter in cells transfected with the empty expression vector (pcDNA3).

## Discussion

Within the TGFβ superfamily, there are a total of seven type I receptors, commonly referred to as ALKs 1–7 [28]. Although individual ligands are known to signal through different type I receptors in different contexts [29], ALK4 was previously considered the only type I receptor used by the activins. Recently, however, activin B and activin AB where shown to signal through ALK7 in a murine pancreatic β cell line [19]. Here, we show that *ALK7*, like *ALK4*, mRNA is expressed in both adult murine pituitary gland and immortalized murine gonadotrope cells, LβT2. Importantly, we further demonstrate that this receptor can broker activin B, but not activin A, signaling in this cell type. Given that activin B appears to be the physiologically relevant activin family member in the pituitary [8,10], these results may uncover a heretofore unappreciated mechanism of *Fshb *gene regulation.

Despite its clear ability to transduce activin B signals, transfected ALK7 was less effective than ALK4 in potentiating the ligand's effects. It is therefore possible that activin B may have a higher affinity for ALK4 than for ALK7. Unfortunately, because the type II receptors are the high affinity binding sites for activins, it is difficult to directly assess relative type I receptor affinities [30]. Nonetheless, our data are consistent with the notion that affinity differences might be involved. Not only does wild-type ALK4 potentiate activin B signaling to a greater extent than wild-type ALK7, but the kinase-deficient form of ALK4 is also more efficient in inhibiting both endogenous (basal) and exogenous activin B signaling. Nonetheless, because the constructs used here had different epitope tags, we were unable to measure relative levels of expression of the two receptors and therefore cannot rule out the possibility that expression level contributed to the observed results. However, constitutively active ALK7(TD) was equivalent to or more potent than ALK4(TD) in stimulating endogenous *Fshb *expression and *Fshb *promoter activity. Given that all three ALK7 constructs (wild-type, KR, and TD) were created in the same expression vector, we do not believe that lower ALK7 wild-type or KR receptor expression completely accounts for their lesser effects on activin B-mediated signaling.

Mechanisms whereby ALK4 and ALK7 regulate *Fshb *promoter activity appear to be conserved. We and others noted previously that depletion of intracellular Smad3 protein levels by RNA interference inhibits, but does not completely block, ALK4(TD)- or activin A-dependent regulation of *Fshb *[3,22,31]. The same pattern of results is observed here with ALK7(TD). These data suggest that activin B signaling via ALK4 or ALK7 can use both Smad3-dependent and -independent mechanisms to regulate *Fshb *transcription [32,33].

Previous experiments indicated that the effects of activin AB were also augmented by transfected ALK7 [19] and we observed the same pattern of results in our analyses (data not shown). Given that activin AB is a heterodimer of the INHBA and INHBB subunits, it is not immediately obvious how ALK7 could mediate its response. Activin A (an INHBA homodimer) appears incapable of binding this receptor (or does so only weakly [24]), so how is it that in the context of the activin AB heterodimer, the INHBA subunit acquires the ability to bind to one of the two ALK7 proteins contained within the receptor complex? Perhaps endogenous ALK4 expressed within the cell lines is sufficient to partner with exogenous ALK7; alternatively, conformational changes in the INHBA subunit, when partnered with INHBB, may allow it to interface more efficiently with ALK7. The results of future crystallographic and mutagenic studies will no doubt clarify the basis for specificity and flexibility in activin/type I receptor interactions.

ALK2, also known as ActRIA or ACVR1, was initially characterized as an activin type I receptor based on its ability to bind iodinated activin A in transfection/binding studies [17]. However, subsequent investigations failed to show that ALK2 was capable of propagating activin A signals [15] and here we similarly see that this receptor does not transduce activin A or B signals to the *Fshb *promoter. In fact, ALK2 over-expression actually inhibits the actions of both ligands, likely because it can bind them but not propagate their signals. In this way, ALK2 can function as a dominant-negative regulator of activin action in a manner analogous to that observed with kinase-deficient forms of ALK4 and ALK7. Interestingly, though not directly involved in activin signaling, ALK2 is expressed in LβT2 cells and may play a role in *Fshb *transcription by transducing BMP signals [9].

Although ALK7, like ALK4, can clearly propagate activin B signals, we have yet to demonstrate a physiological role for this receptor in *Fshb *regulation. Whereas *ALK7 *mRNA is expressed in LβT2 cells and in the adult pituitary, we must confirm its expression in gonadotropes of the latter. Even if expressed in these cells, it is not clear that it will be required for FSH synthesis as *ALK7*^-/- ^mice are both viable and fertile [34]. *ALK4 *is expressed in gonadotropes [5,35,36] and can mediate activin B signaling in this system as shown here. Therefore, ALK4 could compensate for the loss of ALK7 in these and other cells [34].

## Conclusion

Collectively, the data suggest that while ALK7 *can *propagate activin B signals to regulate *Fshb *promoter activity, ALK4 may be the preferred receptor for this ligand, as it is for activin A. Ultimately, ablation of *ALK4 *and *ALK7 *alone and together within gonadotropes will be necessary to determine their relative roles in mediating activin B-regulated FSH synthesis *in vivo*.

## Competing interests

The author(s) declare that they have no competing interests.

## Authors' contributions

DJB designed the study, performed many of the transfection experiments and analyses, and drafted the manuscript. KBL measured the effects of constitutively active receptors on endogenous gene expression. MMS performed the Western blot analyses. All authors read and approved the final manuscript.
